# The Influence of the Dietary Lipid Level on Growth Performance, Lipid Metabolism, Oxidative Response and Hepatopancreatic Health in *Macrobrachium rosenbergii*

**DOI:** 10.3390/ani15192818

**Published:** 2025-09-26

**Authors:** Haoyue Guo, Jie Xu, Kangyu Deng, Anran Wang, Chungui Huang, Min Zhang, Deng Deng, Huangen Chen, Shuyan Miao

**Affiliations:** 1 College of Animal Science and Technology, Yangzhou University, 48 Wenhui East Road, Yangzhou 225009, China; guohaoyue1001@163.com (H.G.); xujie0301@126.com (J.X.); anranw@outlook.com (A.W.); 2 Shenzhen Alpha Feed Co., Ltd., 10E, Haiwang Building, Nanhai Avenue, Nanshan District, Shenzhen 518054, China; dengky2025@163.com (K.D.); ddengq@126.com (D.D.); 3 Jiangsu Fishery Technology Promotion Center, 302 Hanzhongmen Street, Gulou District, Nanjing 210003, China; chunguihuang@126.com (C.H.); minizh2025@163.com (M.Z.); huanggenchen@163.com (H.C.)

**Keywords:** dietary lipid, growth performance, lipid metabolism, oxidative response, hepatopancreatic health, *Macrobrachium rosenbergii*

## Abstract

This study examined how the dietary lipid levels influence growth performance, lipid metabolism, oxidative response and hepatopancreatic health in *Macrobrachium rosenbergii*. A total of 720 post-larvae (0.86 ± 0.01 g) were divided into 4 groups according to the lipid levels in 43% protein-contained diets: 6% (L6), 8% (L8), 10% (L10), and 12% (L12). The results exhibited a significant increase in both the weight gain rate (WGR) and specific growth rate (SGR) in the L8 group (*p* < 0.05). Hepatopancreatic lipid metabolism genes (*fas*, *acc*, *srebp1*, and *fabp*) showed increased expression at higher dietary lipid levels (*p* < 0.05). The expression of *ampk* was significantly reduced, whereas the expression of *atgl* was increased in the L8 group (*p* < 0.05). Additionally, the activities of total superoxide dismutase (SOD), glutathione S-transferase (GST), and total antioxidant capacity (T-AOC) level were significantly higher in the L8 group, while the content of malondinaldehyde (MDA) was significantly reduced (*p* < 0.05). H&E staining of the hepatopancreas revealed that high-lipid diets resulted in severe hepatopancreas damage. Moreover, the L8 group exhibited consistently high cumulative survival rates under both ammonia nitrogen and high-temperature stress (*p* < 0.05). In conclusion, this study recommends a dietary lipid level of 8% to optimize growth performance in *M. rosenbergii* under high-density rearing conditions (70 prawns/m^3^).

## Simple Summary

Although the input of high-fat feed is a nutritional guarantee for the good growth of prawns under high-density farming, excessive lipid intake may have a negative impact on the hepatopancreas health. This study aims to investigate the effects of dietary lipid levels on the growth, oxidative stress and hepatopancreatic health of *Macrobrachium rosenbergii* under high-density rearing conditions (70 prawns/m^3^). The results of this research suggest that a dietary lipid level of 8% is beneficial for promoting growth rates, increasing antioxidant capabilities, stimulating the immune system, and preserving the physiological homeostasis of prawns; however, excessive lipid levels have been shown to adversely affect hepatopancreatic functionality by inhibiting the degradation and transportation of lipids. These findings provide data support for the precise nutritional requirements and technical guidance of efficient breeding of *Macrobrachium rosenbergii*.

## 1. Introduction

*Macrobrachium rosenbergii* is a key aquaculture species in China, prized for its palatable and nutrient-rich meat [1]. Annual production of *M. rosenbergii* has increased steadily, indicating its substantial market demand and economic value. It is evident that *M. rosenbergii* farmers aim to increase profitability by decreasing breeding costs and increasing breeding output through the optimization of feed formulations and increases in feed conversion rates [2,3].

Aquatic animals rely heavily on lipids as essential nutrients, which play indispensable roles in various physiological processes [4]. Under these circumstances, lipid intake has emerged as a critical area for exploration [5]. Appropriate lipid intake provides necessary energy and nutrition [6], improves feed palatability and increases feed consumption [7], potentially resulting in greater farming benefits. However, significant discrepancies exist in the current research regarding the optimal dietary lipid requirement of *M. rosenbergii*. Specifically, the optimal lipid requirement for the postlarval stage of this prawn is reported to be 8–10% [8,9], while that for the adult stage ranges from 6 to 9% [10,11]. Generally, the requirement of dietary lipid depends on many factors, including the culture models and water quality indexes, which make precision nutrition research necessary. Jiangsu, as the main production area, adopts a unique high-density aquaculture model. Taking Gaoyou area as an example, the traditional pond aquaculture density reaches 1.05 to 1.8 million prawns per hectare (about 70 to 120 prawns/m^3^), which is much higher than that of production areas such as Guangdong and Thailand (typically 50 prawns/m^3^) [12]. To maintain rapid growth under high-density stress, high-protein nutrient-intensive feed is commonly used in aquaculture. In our previous research, it was demonstrated that the appropriate protein level in high-density mode is 42.5%. Excessive lipid intake amid high-density rearing stress may adversely affect prawn health, inducing oxidative stress and hepatopancreatic damage [13,14]. In this mode, the precise lipid requirement of *M. rosenbergii* become the focus of our research.

This study therefore investigated the effects of dietary lipid levels on the growth, oxidative stress, and hepatopancreatic health in prawns reared with high-density conditions (70 prawns/m^3^ was suggested), to provide data support for the precise nutritional requirements for the high efficiency of prawn culture.

## 2. Materials and Methods

### 2.1. Diets of the Experimental Groups

The ingredient formulation and nutritional profile of diets are provided in Table 1, and the fatty acid content of the diets is shown in Table 2. The primary protein sources included fish meal, soybean meal, poultry by-product meal, shrimp meal, and peanut meal, while the lipid components were supplied by soybean and fish oil. Four experimental groups were fed diets with different lipid levels: 6% (L6), 8% (L8), 10% (L10) and 12% (L12). All components were crushed and sieved through a 100 μm mesh, and then fully blended with the oil mixture. Then, it was made using a feed pellet machine (South China University of Technology, Guangzhou, China, F-26), dried in a ventilated oven at 45 °C, and stored at −20 °C until use [15].

### 2.2. Experimental Design and Management of Animals

The experimental *M. rosenbergii* were provided by the Sufeng Shrimp Factory in Yangzhou City. Before the feeding trial, all prawns were reared in a cement tank inside a greenhouse for a temporary period of 2 weeks. A total of 720 prawns (0.86 ± 0.01 g) were randomly divided into four groups. Each group contained four replicates, and each replicate contained 45 prawns reared in a nylon mesh cage. Prawns were provided with feed twice per day, at 06:30 and 18:30, until they reached satiation. A daily water exchange ranging from 30% to 50% was carried out. Throughout the period, water temperature fluctuated between 25 and 31 °C, while pH values remained within 7.8–8.0. Dissolved oxygen concentrations were kept above 5 mg/L, nitrite nitrogen levels did not exceed 0.1 mg/L, and total ammonia nitrogen was maintained below 0.2 mg/L.

### 2.3. Specimen Collection and Examination

The prawns were fasted for 24 h after the feeding trial, counted, and weighed. The growth index of prawn was calculated according to the formula listed in 2.6 [16].

For each of the four replicate cages per group, muscle tissue specimens from six prawns per cage were stored at −20 °C to maintain biochemical integrity for subsequent compositional analysis using the AOAC standard method [17]. In this experiment, the content of fatty acids in diets was analyzed by using high performance gas chromatography combined with mass spectrometry (Agilent Technologies, Santa Clara, CA, USA, GC-MS). First, prepare the standard solution, and then extract the fatty acids from the feed. Take 200 mg of the feed sample into a 2 mL centrifuge tube, then add 1 mL of chloroform solution, and extract for 30 min in an ultrasonic environment. Then take the supernatant and add 2 mL of 1% sulfuric acid-methanol solution, add 1 mL of n-hexane, and place it in an 80 °C water bath for extraction for 30 min. Finally, wash with pure water. Finally, 25 μL of methyl salicylate was added as the internal standard, mixed well and then added to the injection bottle for GC-MS detection.

Furthermore, hepatopancreatic samples were collected from five prawns and stored in liquid nitrogen for RNA extraction. We designed the gene-specific primers using Primer Express software v3.0.1 (Table 3). The *β-actin* gene functioned as the internal reference. Quantitative analysis of transcriptional levels was conducted through real-time PCR, with data normalization and relative quantification performed according to the 2^−ΔΔCt^ computational model [18].

Haemolymph samples were collected from the pericardial sinuses of the prawns. The haemolymph was mixed with an anticoagulant (Sodium citrate 13.2 g/L, citric acid 4.8 g/L, glucose 14.7 g/L) at a 1:1 ratio and then centrifuged to obtain the haemolymph [19].

Commercial kits supplied by Nanjing Jiancheng (China) were used to evaluate various biochemical parameters in the haemolymph and hepatopancreas, including the activities of triglyceride (TG) and total cholesterol (TCHO) in the haemolymph, the activity of glutathione S-transferase (GST), total superoxide dismutase (SOD), glutathione peroxidase (GSH-Px), catalase (CAT), the total antioxidant capacity (T-AOC) level, the content of malondinaldehyde (MDA), as well as the activity of trypsin and lipase in hepatopancreas [20].

### 2.4. Histological Evaluation

The hepatopancreases were fixed in 4% neutral buffered formaldehyde and subsequently processed, sectioned, and stained following established protocols [21]. Histological characteristics were examined using biological microscope (Olympus, Tokyo, Japan, BX53).

### 2.5. Stress Tests

During the feeding trial, samples were collected to assess ammonia nitrogen stress. From each treatment group, 32 prawns (10.59 ± 0.19 g) were randomly allocated into four replicate tanks (10 L; *n* = 8 prawns/replicate). The prawns were exposed to ammonia nitrogen stress. The concentrations, which were determined according to preliminary tests and LC_50_ data, were applied to the prawns for a 48 h period [20]. Survival rates were recorded at 12 h intervals.

Finally, the prawns (10.59 ± 0.19 g) were subjected to acute heat stress at 38.5 °C. Each dose group comprised four replicate groups and 8 prawns/replicate. The prawn mortality rate was assessed at 4, 8, 12, 16, 20, and 24 h.

### 2.6. Statistical Analysis and Calculation Formula

The growth indicators were calculated via the following formula:

Weight gain rate (WGR, %) = 100 × (W_t_ − W_i_)/W_i_

Specific growth rate (SGR, %/d) = 100 × (ln W_t_ − ln W_i_)/t

Hepatosomatic index (HSI, %) = 100 × W_h_/W_t_

Feed conversion ratio (FCR) = F/(W_t_ − W_i_)

Condition factor (CF, %) = 100 × W_t_/L_t_^3^

F: weight of feed consumed (g); W_t_: final mean weight (g); L_t_: final mean length; W_i_: initial mean weight (g); t: duration of the feeding trial (d); W_h_: hepatopancreas weight (g).

SPSS 27.0 software was used for all the analyses, and the test results are presented as the means ± SD of four replicates. One-way ANOVA and Duncan’s tests were used to compare multiple groups [22]. Differences for *p* < 0.05 were considered statistically significant.

## 3. Results

### 3.1. Growth Performance, Feed Utilization, and the Hepatopancreas Index of M. rosenbergii

Different lipid levels had significant effects on multiple growth parameters and feed utilization in *M. rosenbergii* (Table 4). Specifically, prawns in the L8 group exhibited significantly greater FW, WGR, and SGR values (*p* < 0.05). Conversely, the FCR in the L6 group was notably elevated relative to that of the other groups. Additionally, CF of the prawns in the L6 group was significantly lower (*p* < 0.05).

### 3.2. Nutritional Composition of Muscles

Results showed that the data revealed no significant differences in the composition of different lipid levels groups (*p* > 0.05) (Table 5).

### 3.3. Activity of Hepatopancreatic Digestive Enzymes

**Figure 1:**
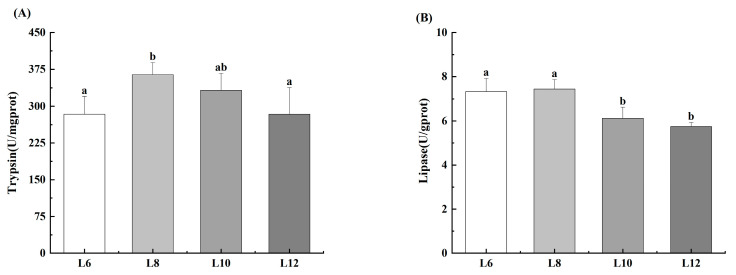
Effects of different lipid levels on hepatopancreas digestive enzymes activities of *M. rosenbergii* (*n* = 4). (**A**) Trypsin activity; (**B**) Lipase activity. Experimental outcomes demonstrate arithmetic means ± SD derived from quadruplicate experimental repetitions (*n* = 4). Distinct alphabetic superscripts identify statistical groupings through Duncan’s multiple range test, where differentiated lowercase characters signify significant divergence (*p* < 0.05). Uniform annotation conventions persist across subsequent data presentations.

Prawns in the L8 group demonstrated significantly higher trypsin activity (*p* < 0.05) (Figure 1A). Specifically, the L10 group exhibited greater trypsin activity than that of the L6 and L12 groups (*p* < 0.05). Additionally, the lipase activity of the prawns in groups L6 and L8 significantly increased (*p* < 0.05) (Figure 1B).

### 3.4. Haemolymphatic Biochemical Indices

**Figure 2:**
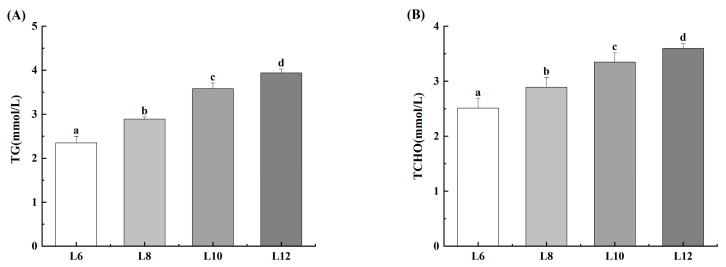
Effects of different lipid levels on hemolymphatic lipid metabolite parameters of *M. rosenbergii* (*n* = 4). (**A**) Triglyceride (TG); (**B**) Total cholesterol (TCHO). Experimental outcomes demonstrate arithmetic means ± SD derived from quadruplicate experimental repetitions (*n* = 4). Distinct alphabetic superscripts identify statistical groupings through Duncan’s multiple range test, where differentiated lowercase characters signify significant divergence (*p* < 0.05). Uniform annotation conventions persist across subsequent data presentations.

As shown in Figure 2, the findings revealed that increasing dietary lipid levels led to significant increases in the activities of TG and TCHO in the haemolymph of the prawns (*p* < 0.05). Notably, the L12 group exhibited the highest levels of both TG and TCHO (*p* < 0.05).

### 3.5. Expression of Lipid Metabolism-Associated Genes

**Figure 3:**
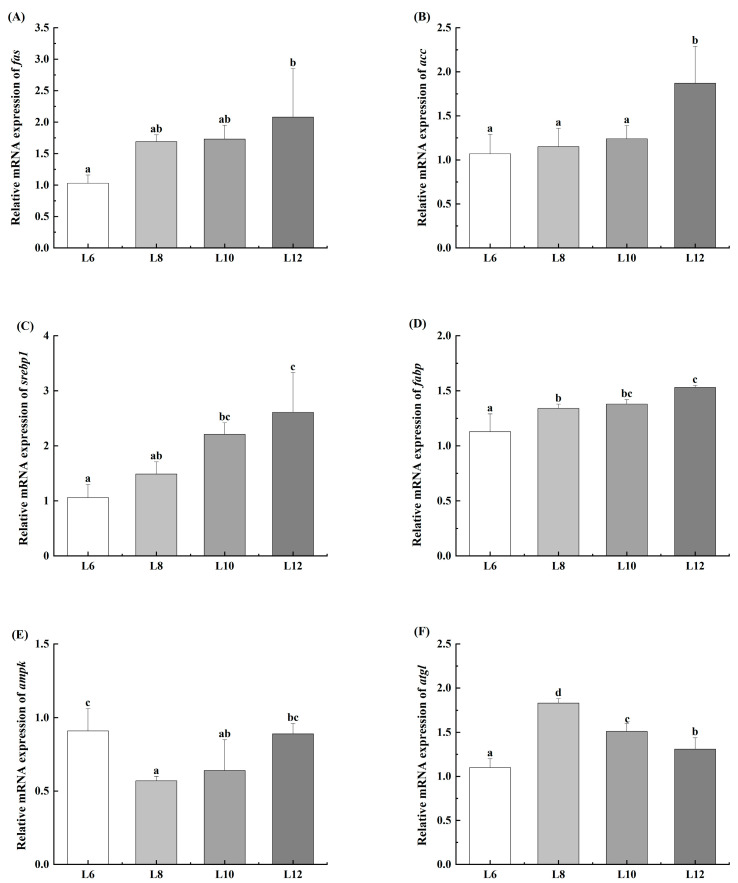
Effects of different lipid levels on relative expression of lipid metabolism in hepatopancreas of *M. rosenbergii* (*n* = 4). (**A**) fatty acid synthase (*fas*); (**B**) acetyl-CoA carboxylase (*acc*); (**C**) sterol regulatory element-binding protein 1 (*srebp1*); (**D**) fatty acid binding protein (*fabp*); (**E**) adenylate activated protein kinase (*ampk*); (**F**) adipose triacylglyceride lipase (*atgl*). Experimental outcomes demonstrate arithmetic means ± SD derived from quadruplicate experimental repetitions (*n* = 4). Distinct alphabetic superscripts identify statistical groupings through Duncan’s multiple range test, where differentiated lowercase characters signify significant divergence (*p* < 0.05). Uniform annotation conventions persist across subsequent data presentations.

As shown in Figure 3, the L12 group demonstrated significantly greater expression levels of *fas*, *acc*, *srebp1*, and *fabp* (*p* < 0.05). With increasing dietary lipid levels, an increasing trend was observed in the expression of *srebp1* and *fabp*, and the expression of *ampk* decreased but then increased. The expression of *ampk* was significantly reduced in the L8 group, whereas the expression of *atgl* was increased (*p* < 0.05).

### 3.6. Antioxidant Capacity

**Figure 4:**
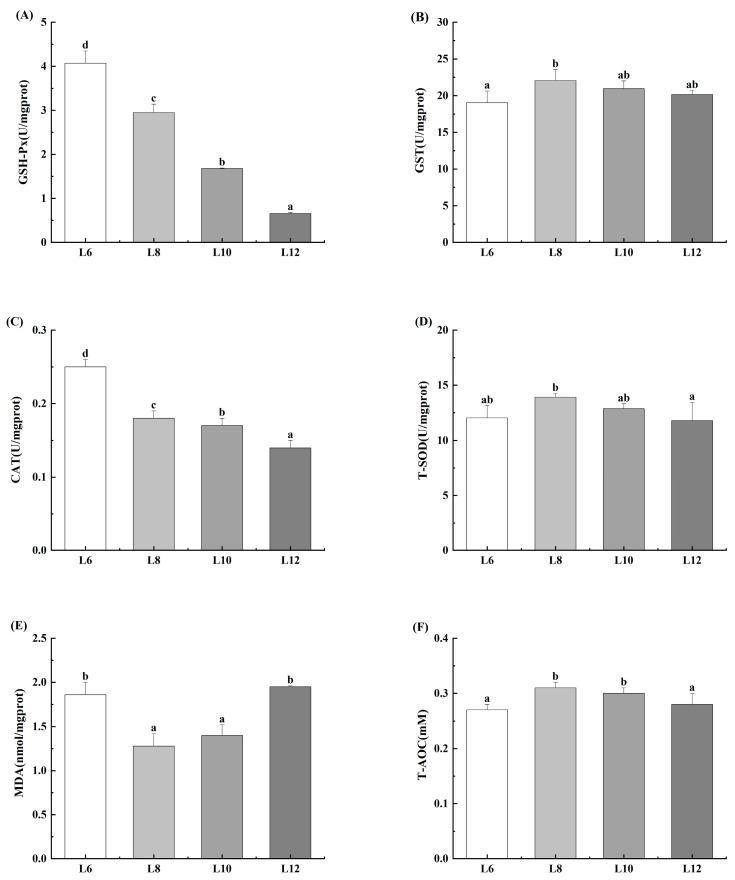
Effects of different lipid levels on hepatopancreas antioxidant capacity of *M. rosenbergii* (*n* = 4). (**A**) Glutathione peroxidase (GPx); (**B**) Glutathione S-transferase (GST); (**C**) catalase (CAT); (**D**) Total superoxide dismutase (T-SOD); (**E**) Malondinaldehyde (MDA); (**F**) Total antioxidant capacity (T-AOC). Experimental outcomes demonstrate arithmetic means ± SD derived from quadruplicate experimental repetitions (*n* = 4). Distinct alphabetic superscripts identify statistical groupings through Duncan’s multiple range test, where differentiated lowercase characters signify significant divergence (*p* < 0.05). Uniform annotation conventions persist across subsequent data presentations.

As shown in Figure 4, the L12 group exhibited a statistically significant reduction in the activities of GSH-Px and CAT (*p* < 0.05). Additionally, the activities of GST and T-SOD were significantly greater in the L8 group (*p* < 0.05). Furthermore, the content of MDA was significantly lower in both the L8 and L10 groups (*p* < 0.05). Finally, T-AOC level was significantly increased in the L8 and L10 groups (*p* < 0.05).

### 3.7. Histological Examination

**Figure 5:**
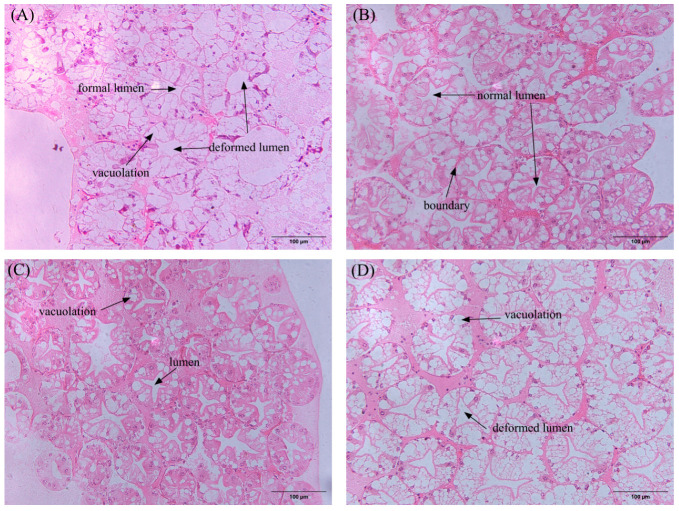
H&E-staining hepatopancreatic tissue sections of prawns under varying lipid levels (magnification × 100). (**A**) 6% lipid diet; (**B**) 8% lipid diet; (**C**) 10% lipid diet; (**D**) 12% lipid diet. Lumen: Undigested lipid accumulates in the lumen of the hepatic tubules, compressing the structure of the glandular ducts and causing deformation of the lumen. Vacuolation: Triglycerides abnormally accumulate in hepatopancreas cells, forming lipid droplet vacuoles. Boundary: The boundary of a healthy hepatopancreas is a sharp and clear valve-like structure.

H&E staining revealed that the lumen of hepatopancreas in L12 group was significantly enlarged, star-shaped lumen deformed and vacuolated. In contrast, while the hepatopancreas tissues of the L6 and L10 groups showed partial vacuolation, their overall tissue structure remained well-defined and intact, with relatively normal hepatopancreatic morphology and structure. Notably, the hepatopancreas tissues in the L8 group displayed normal star-shaped lumens with clear boundaries and tightly arranged structures, indicating superior preservation of hepatopancreatic morphology and structure (Figure 5).

### 3.8. Cumulative Survival Rate

**Figure 6:**
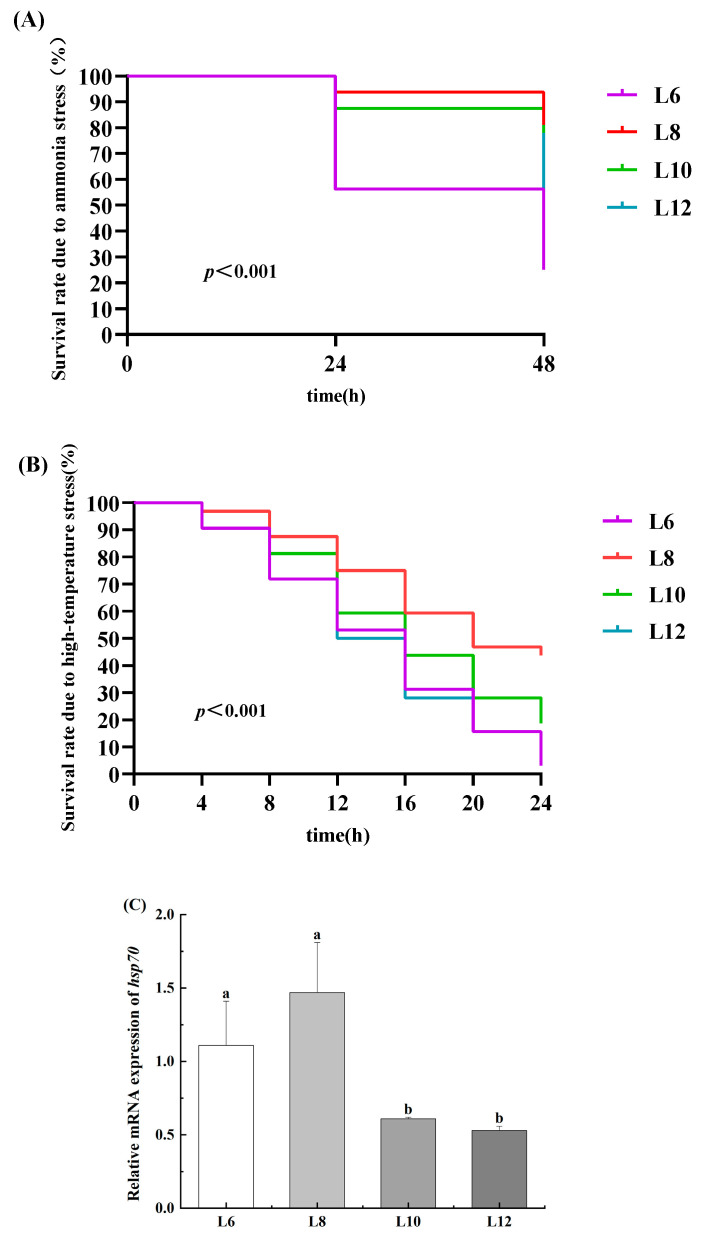
Impact of dietary lipid levels on *M. rosenbergii* survival under ammonia-N and high-temperature stress (*n* = 4). (**A**) Effects of Ammonia-N stress on the lifespan of *M. rosenbergii*; (**B**) Effects of high-temperature stress on the lifespan of *M. rosenbergii*; (**C**) Expression of *hsp70* in hepatopancreas of *M. rosenbergii*. Experimental outcomes demonstrate arithmetic means ± SD derived from quadruplicate experimental repetitions (*n* = 4). Distinct alphabetic superscripts identify statistical groupings through Duncan’s multiple range test, where differentiated lowercase characters signify significant divergence (*p* < 0.05). Uniform annotation conventions persist across subsequent data presentations.

The survival curve of *M. rosenbergii* individuals that were subjected to ammonia nitrogen stress demonstrated notable variations among prawns that were fed different levels of lipids over the 48 h period. The cumulative survival rates of the prawns in the L8 and L10 groups were lower (Figure 6A). The 24-hour mortality rate of prawns under ammonia nitrogen stress in L6 group was significantly increased, while the 48-hour mortality rates of prawns in L8 and L10 group were significantly lower than those in the other groups (*p* < 0.05) (Table 6).

The cumulative survival rate of the prawns in the L8 group was clearly higher under high-temperature stress (Figure 6B). The 8-hour mortality rate of prawns in L8 and L10 group was significantly reduced under high-temperature stress (*p* < 0.05). The 20-hour mortality rate of prawns in L8 group was significantly lower than that in the other groups, while the mortality rates of prawns in L6 and L12 group were significantly increased (*p* < 0.05) (Table 7). Moreover, the expression levels of *hsp70* were significantly elevated in the L6 and L8 groups (*p* < 0.05) (Figure 6C).

## 4. Discussion

The rapid advancement of aquaculture and the increasing use of precision feeding technology have made reducing feed waste and enhancing environmental sustainability key priorities for many farmers [23]. Precision feeding not only optimizes feed lipid levels but also offers innovative solutions for sustainable farming practices and improved economic returns [24]. The hepatopancreas is a critical digestive gland and immune organ [25,26]. High-fat feeding induces significant lipid accumulation in the hepatopancreas [14,27], leading to steatosis that not only disrupts the organ’s normal structure and function but also can cause cellular dysfunction and apoptosis [28,29]. Conversely, a low-fat diet may not provide enough energy, leading to possible atrophy and cell dysfunction in the hepatopancreas [4]. H&E staining revealed lipid-level-associated vacuolization in the hepatopancreas. This may lead to a series of negative effects, including decreased hepatopancreas immunity [30,31], and digestive function in the prawns.

Above all, antioxidant enzyme activity could be suppressed [32], resulting in the accumulation of free radicals and peroxides within the cells, which in turn trigger immune-regulatory dysfunction [33,34]. GPX and CAT play crucial roles as antioxidant enzymes [35]. Although CAT and GPX participate in different pathways to catalyze the decomposition of hydrogen peroxide, and they work together to maintain the intracellular redox balance [36]. The elevated lipid levels in feed resulted in trends of decreasing GPX and CAT activity; however, this does not mean that lower feed lipid contents can avoid causing hepatopancreatic injury. Moreover, the increase in the GST activity indicated an improvement in the detoxification function of the hepatopancreas [37]. However, the decrease in GST activity suggests that a low-fat diet could cause a decrease in the detoxification function of the hepatopancreas. The antioxidant enzymes of an organism, namely, SOD, GPX and CAT, work in coordination with each other [38,39]. Hydrogen peroxide that is generated by the disproportionation of superoxide anions by SOD can be further decomposed by GPX and CAT, thus eliminating hydrogen peroxide [40]. In addition, hepatopancreatic injury may lead to a decreased T-AOC, reflecting an overall decrease in antioxidant capacity [41,42].

Under intensive *M. rosenbergii* aquaculture, ammonia nitrogen generated from the decomposition of uneaten feed, feces, and organic matter can easily accumulate to a toxic concentration in the water [43]. In May and June, which is a period of rapid development, water temperatures frequently exceed 33–35 °C, exacerbating the effects of high stocking densities. The mortality rates observed in both anti-stress tests indicated that hepatopancreatic injury may reduce immunity and decrease resistance to environmental factors.

Generally, hepatopancreatic injury can lead to an imbalance in lipid synthesis, degradation and transport [44]. As the extent of hepatopancreatic injury worsened, an increase in the serum contents of TG and TCHO were observed. During hepatopancreatic injury, the reduced expression of *atgl* may exacerbate lipid metabolism disorders. In addition, the reduced expression of *fabp* may impair fatty acid transport and triglyceride degradation. In summary, feeding high-fat diet leads to hepatopancreas injury in prawns, which affects the decomposition ability of lipids in the hepatopancreas [45].

Additionally, the hepatopancreas’s capacity to secrete digestive enzymes is also influenced by hepatopancreas injury [46]. The enhanced trypsin activity suggests that moderate lipid supplementation may stimulate digestion through lipid-mediated regulation of hepatopancreatic secretion [47]. However, excess lipids may have an inhibitory effect on the hydrolase system, possibly due to oxidative stress caused by lipid overload [48]. However, lipase synthesis capacity is saturated or negatively feedback regulated under high lipid intake [49]. This result is similar to that study in *Litopenaeus vannamei*, where high-fat diets promoted hepatopancreatic lipid accumulation, increased inflammatory responses, and inhibited mitochondrial autophagy [14]. Notably, the reduction in enzyme output at slightly higher lipid levels emphasizes the physiological limits of lipid utilization in crustaceans, which may be related to endoplasmic reticulum stress or mitochondrial dysfunction in response to excess lipid supply [50].

The content of all fatty acids in L10 and L12 has increased significantly, and the negative effects of some of these fatty acids will be magnified. The content of C18:2 in group L12 was as high as 34.65 g/kg, which was a huge potential source of oxidative stress. Polyunsaturated fatty acids are highly prone to lipid peroxidation due to the presence of multiple unsaturated double bonds [51], which can lead to the formation of harmful substances. These peroxides can attack the cell membrane, causing damage to mitochondrial function and inducing apoptosis or necrosis of cells [52]. Although the C20:4 content in the feed is not high, a high level of C18:2 can be used as a substrate to be converted into ARA, which may aggravate the inflammatory response and damage the hepatopancreas tissue [53]. Previous studies have shown that C22:1 is toxic to the heart and hepatopancreas [54]. The content of C22:1 in this feed increased from 1.95 g/kg in group L6 to 8.64 g/kg in group L12. A high level of C22:1 is very likely to be an important factor leading to hepatopancreatic injury. The accumulation of excessive free saturated fatty acids within cells can cause lipid toxicity damage to cells [55] and increase the metabolic pressure on the hepatopancreas, and the content of C16:0 in the L12 group is higher than that in other groups. In conclusion, the negative impact of fatty acid composition in feed on the hepatopancreas is consistent with the results of our previous detection indicators.

## 5. Conclusions

Overall, the results of this research suggest that a dietary lipid level of 8% is beneficial for promoting growth rates, increasing antioxidant capabilities, stimulating the immune system, and preserving the physiological homeostasis of prawns. Moreover, it is recommended that the dietary lipid levels for *M. rosenbergii* do not surpass 10%, as excessive levels have been shown to adversely affect hepatopancreatic functionality by inhibiting the degradation and transportation of lipids.

## Figures and Tables

**Table 1:** Nutritional formulation and chemical composition profiles of study diets (% DM).

Ingredients (g/100 g)	Groups			
	L6 (6%)	L8 (8%)	L10 (10%)	L12 (12%)
Soybean meal-43%	23.00	23.00	23.00	23.00
Fish meal-Imported Japanese grade-70%	16.00	16.00	16.00	16.00
Fish meal-Domestic semi-skimmed-65%	12.00	12.00	12.00	12.00
Shrimp meal-61%	7.50	7.50	7.50	7.50
Peanut meal-53%	5.00	5.00	5.00	5.00
Poultry by-product meal-65%	2.00	2.00	2.00	2.00
Squid paste	2.00	2.00	2.00	2.00
Wheat flour	19.00	19.00	19.00	19.00
Shrimp paste	2.00	2.00	2.00	2.00
Soybean lecithin	2.50	2.50	2.50	2.50
Ca(H_2_PO_4_)_2_	1.50	1.50	1.50	1.50
Soybean oil	0.00	1.00	2.00	3.00
Fish oil	0.00	1.00	2.00	3.00
Choline chloride-60%	0.25	0.25	0.25	0.25
Vitamin premix ^1^	0.20	0.20	0.20	0.20
Mineral premix ^2^	0.20	0.20	0.20	0.20
Vitamin C—phosphatidic	0.10	0.10	0.10	0.10
Organic acid	0.05	0.05	0.05	0.05
Bentonite	6.50	4.50	2.50	0.50
Guar gum	0.20	0.20	0.20	0.20
Total	100.00	100.00	100.00	100.00
Proximate composition (% dry matter)				
Moisture	9.73	9.29	9.44	9.59
Crude protein	42.81	43.19	43.06	43.00
Crude lipid	5.94	8.06	9.97	12.14
Ash content	16.00	14.14	12.24	10.52

^1^ Vitamin mixture (IU or g/kg): thiamine, 0.5 g; riboflavin, 0.7 g; cyanocobalamin, 0.002 g; menadione, 0.5 g; retinol, 450,000 IU; pyridoxine HCl, 0.6 g; cholecalciferol, 150,000 IU; vitamin E, 5 g; nicotinic acid, 3.5 g; folic acid, 0.15 g; biotin, 0.060 g. ^2^ Mineral mixture (g/kg): Calcium phosphate monobasic: 10; Magnesium sulfate heptahydrate: 2.4; Potassium chloride: 4.5; Sodium chloride: 2.1; Ferrous sulfate monohydrate: 0.155; Cupric sulfate pentahydrate: 0.040; Zinc sulfate monohydrate: 0.080; Manganese sulfate monohydrate: 0.030; Potassium iodide: 0.0117; Cobalt chloride hexahydrate: 0.0048; Sodium selenite: 0.0024.

**Table 2:** Composition of feed fatty acids (% total fatty acid methyl esters).

Content (g/kg)	L6	L8	L10	L12
C14:0	2.25	2.98	3.71	4.44
C16:0	10.05	12.88	15.71	18.54
C16:1	2.41	3.41	4.41	5.41
C18:0	2.41	3.18	3.95	4.72
C18:1	12.02	15.39	18.76	22.13
C18:2	18.39	23.81	29.23	34.65
C18:3	3.06	3.88	4.70	5.52
C20:1	1.73	3.26	4.79	6.32
C20:4	0.77	0.80	0.83	0.86
C22:1	1.95	4.18	6.41	8.64
C22:5	0.67	0.75	0.83	0.91
C22:6	2.47	3.32	4.17	5.02
Sum	58.17	77.83	97.49	117.15

**Table 3:** Oligonucleotide primer sequences employed for RT-qPCR amplification of target genes.

Primer Name	Position	Primer Sequence	Length
*fas*	F (5′-3′)	TCACTTCTCAACACCCAATCCA	22
	R (5′-3′)	TTGCAGACCGAAGAAGGACG	20
*acc*	F (5′-3′)	GATGAGGGATTCAAGCCCAGTT	22
	R (5′-3′)	TCCCTGTCTTCACCCCACGA	20
*srebp-1*	F (5′-3′)	CAGACACTGGCCGAGATGTG	20
	R (5′-3′)	GAGCTGGAGCATGTCTTCGAT	21
*fabp*	F (5′-3′)	AACGACGAATGGACGCTGAA	20
	R (5′-3′)	TTCCCTTAGTGGCGTTCTGG	20
*ampk*	F (5′-3′)	TGGAAAGTGAGCATTGACGAAG	22
	R (5′-3′)	CATTGGGGTCACGCAACAGA	20
*atgl*	F (5′-3′)	TTGTATCGCTTTGCCCGTAT	20
	R (5′-3′)	CAAAGGGTAAGACATAAGGCAAT	23
*hsp70*	F (5′-3′)	TGACAAGGGTCGCCTCAGTA	20
	R (5′-3′)	CATTATCTTGTTGCGATCCTC	21
*β-actin*	F (5′-3′)	TCCGTAAGGACCTGTATGCC	20
	R (5′-3′)	TCGGGAGGTGCGATGATTTT	20

**Table 4:** Effects of different lipid levels on the survival, growth performance, and feed utilization of *M. rosenbergii* (*n* = 4).

Treatment	Diets (Lipid Level %)			
	L6 (6%)	L8 (8%)	L10 (10%)	L12 (12%)
IW (g)	0.85 ± 0.01	0.85 ± 0.01	0.86 ± 0.01	0.86 ± 0.01
FW (g)	11.31 ± 0.40 a	12.67 ± 0.32 b	12.42 ± 0.17 b	12.16 ± 0.49 b
WGR (%)	1230.63 ± 48.75 a	1386.03 ± 30.61 c	1340.59 ± 19.62 bc	1306.79 ± 56.87 b
SGR (%/d)	4.17 ± 0.06 a	4.35 ± 0.03 c	4.30 ± 0.02 bc	4.26 ± 0.06 b
HSI (%)	3.46 ± 0.23	2.92 ± 0.36	2.93 ± 0.58	3.29 ± 0.45
FCR	2.82 ± 0.15 b	2.55 ± 0.11 a	2.58 ± 0.07 a	2.59 ± 0.17 a
CF (%)	11.45 ± 0.34 a	12.29 ± 0.24 b	12.14 ± 0.19 b	11.96 ± 0.39 b

Abbreviations: IW, initial individual weight; HSI, hepatosomatic index; FW, final individual weight; SGR, specific growth rate; CF, condition factor; WGR, weight gain rate; FCR, feed conversion ratio. Experimental outcomes demonstrate arithmetic means ± SD derived from quadruplicate experimental repetitions (*n* = 4). Distinct alphabetic superscripts identify statistical groupings through Duncan’s multiple range test, where differentiated lowercase characters signify significant divergence (*p* < 0.05). Uniform annotation conventions persist across subsequent data presentations.

**Table 5:** Effects of different dietary lipid levels on muscle nutritional composition of *M. rosenbergii* (*n* = 4).

	L6 (6%)	L8 (8%)	L10 (10%)	L12 (12%)
Moisture (%)	77.45 ± 0.36	77.53 ± 0.24	77.46 ± 0.74	77.36 ± 0.55
Crude protein (%)	21.04 ± 0.06	21.30 ± 0.24	21.38 ± 0.49	21.24 ± 0.27
Crude lipid (%)	0.83 ± 0.01	0.87 ± 0.04	0.85 ± 0.03	0.84 ± 0.03
Ash (%)	1.38 ± 0.06	1.43 ± 0.08	1.42 ± 0.05	1.41 ± 0.07

Experimental outcomes demonstrate arithmetic means ± SD derived from quadruplicate experimental repetitions (*n* = 4). Distinct alphabetic superscripts identify statistical groupings through Duncan’s multiple range test, where the absence of lowercase letters indicated no significant difference (*p* > 0.05). Uniform annotation conventions persist across subsequent data presentations.

**Table 6:** Effects of dietary lipid levels on survival of *M. rosenbergii* under ammonia-N stress (*n* = 4).

	L6 (6%)	L8 (8%)	L10 (10%)	L12 (12%)
24 h mortality rate (%)	43.75 ± 7.22 ^b^	6.25 ± 7.22 ^a^	12.50 ± 0.00 ^a^	6.25 ± 7.22 ^a^
48 h mortality rate (%)	75.00 ± 10.21 ^c^	18.75 ± 7.22 ^a^	21.88 ± 6.25 ^a^	50.00 ± 17.68 ^b^

Experimental outcomes demonstrate arithmetic means ± SD derived from quadruplicate experimental repetitions (*n* = 4). Distinct alphabetic superscripts identify statistical groupings through Duncan’s multiple range test, where differentiated lowercase characters signify significant divergence (*p* < 0.05). Uniform annotation conventions persist across subsequent data presentations.

**Table 7:** Effects of dietary lipid levels on survival of *M. rosenbergii* under high-temperature stress (*n* = 4).

	L6 (6%)	L8 (8%)	L10 (10%)	L12 (12%)
4 h mortality rate (%)	9.38 ± 6.25	3.13 ± 6.25	3.13 ± 6.25	9.38 ± 6.25
8 h mortality rate (%)	28.13 ± 6.25 ^b^	12.50 ± 0.00 ^a^	18.75 ± 7.22 ^a^	28.13 ± 6.25 ^b^
12 h mortality rate (%)	46.88 ± 6.25 ^b^	25.00 ± 10.21 ^a^	40.63 ± 6.25 ^b^	50.00 ± 10.21 ^b^
16 h mortality rate (%)	68.75 ± 7.22 ^b^	40.63 ± 11.97 ^a^	56.25 ± 7.22 ^b^	71.88 ± 6.25 ^c^
20 h mortality rate (%)	84.38 ± 6.25 ^c^	53.13 ± 6.25 ^a^	71.88 ± 6.25 ^b^	84.38 ± 6.25 ^c^
24 h mortality rate (%)	96.88 ± 6.25 ^c^	56.25 ± 7.22 ^a^	81.25 ± 7.22 ^b^	93.75 ± 7.22 ^c^

Experimental outcomes demonstrate arithmetic means ± SD derived from quadruplicate experimental repetitions (*n* = 4). Distinct alphabetic superscripts identify statistical groupings through Duncan’s multiple range test, where differentiated lowercase characters signify significant divergence (*p* < 0.05). Uniform annotation conventions persist across subsequent data presentations.

## Data Availability

Data will be supplied when requested.
